# Human papillomavirus-related multiphenotypic sinonasal carcinoma: a clinico-pathological dilemma case report

**DOI:** 10.11604/pamj.2021.39.78.26043

**Published:** 2021-05-26

**Authors:** Nouha Ben Abdeljelil, Samiha Mabrouk, Souheil Khalfaoui, Mahdi Farjaoui, Ahlem Bellalah, Abdefattah Zakhama, Rim Hadhri

**Affiliations:** 1 Department of Pathology, Fattouma Bourguiba University Hospital, Monastir, Tunisia,; 2 Department of Neurosurgery, Fattouma Bourguiba University Hospital, Monastir, Tunisia,; 3 Department of Otorhinolaryngology, Fattouma Bourguiba University Hospital, Monastir, Tunisia

**Keywords:** Human papillomavirus, carcinoma, sinonasal tract, immunohistochemistry, case report

## Abstract

Human papillomavirus (HPV)-related multiphenotypic sinonasal carcinoma (HMSC), is a new entity that is restricted to the sinonasal tract and is associated with high-risk HPV. This tumor is suggested to have an indolent behavior with a better prognosis than other carcinomas. We report a unique case of HMSC with a locally aggressive behavior. It is about a 61-year-old men presented with 12 months of unilateral progressive olfactory dysfunction accompanied by exophthalmia of the left eye, declining vision and headaches for 6 months. Computed tomography imaging revealed a voluminous mass occupying the ethmoid, maxillary and frontal sinus with bony destruction of the left ethmoidal blade. Histology showed a tumor composed of variably sized nests, separated by thick mucoid stroma. Tumor cells are plasmacytoid with hyperchromatic nuclei and frequent mitoses. Immunohistochemistry revealed that these cells were positive for cytokeratin AE1/AE3, p16 and negative for CK7, CK20, CD117, p40, p63, S100, synaptophysin and chromogranin.

## Introduction

Human papillomavirus (HPV) is an important causative factor in approximately 20% to 25% of head and neck carcinomas overall. The sinonasal tract represents the second anatomic hot spot from which HPV-related tumors can arise [1]. HPV-related multiphenotypic sinonasal carcinoma (HMSC), originally known as HPV-related carcinoma with adenoid cystic carcinoma (ACC)-like features, is a new entity that is restricted to the sinonasal tract [2]. This tumor firstly described by Bishop *et al*. in 2013, displays features of both a surface-derived carcinoma and a salivary gland carcinoma, particularly ACC, and is associated with high-risk HPV, specifically HPV type 33 [3]. Currently, HMSC is a provisional entity in the most recent WHO classification of head and neck tumors [4].

The largest case series of HMSC is described by Bishop *et al*. who first suggested that despite high-grade histologic appearance, HMSC paradoxically behaves in a relatively indolent manner with frequent local recurrences but only rare metastases and no reported tumor-related deaths [1]. Other reported cases after this series have followed this trend [2,4-9]. Herein, we present a unique case of HMSC with locally aggressive behavior.

## Patient and observation

**Patient information:** we present the case of a 61-year-old men presented with 12 months of unilateral progressive olfactory dysfunction accompanied by exophthalmia of the left eye, declining vision and headaches for 6 months.

**Clinical findings:** physical examination showed left anosmia, non-axil and non-reducible exophthalmia with extrinsic paralysis III.

**Figure 1:**
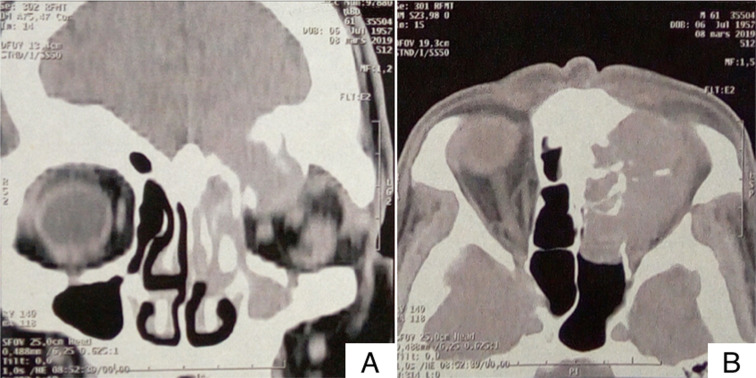
A) axial; B) coronal view of computed tomography showing voluminous spontaneously hyperdense mass taking weak contrast, occupying the ethmoid, maxillary and frontal sinus with bony destruction of the left ethmoidal blade and the roof of the left orbit

**Diagnostic assessment:** computed tomography imaging of the paranasal sinuses revealed a voluminous spontaneously hyperdense mass taking weak contrast, occupying the ethmoid, maxillary and frontal sinus with bony destruction of the left ethmoidal blade and the roof of the left orbit. The mass also invaded the anterior skull base and the conical fat of the orbit with superior rectus muscle causing repression of the left eyeball (Figure 1). The patient subsequently underwent endoscopic nasal biopsy.

**Figure 2:**
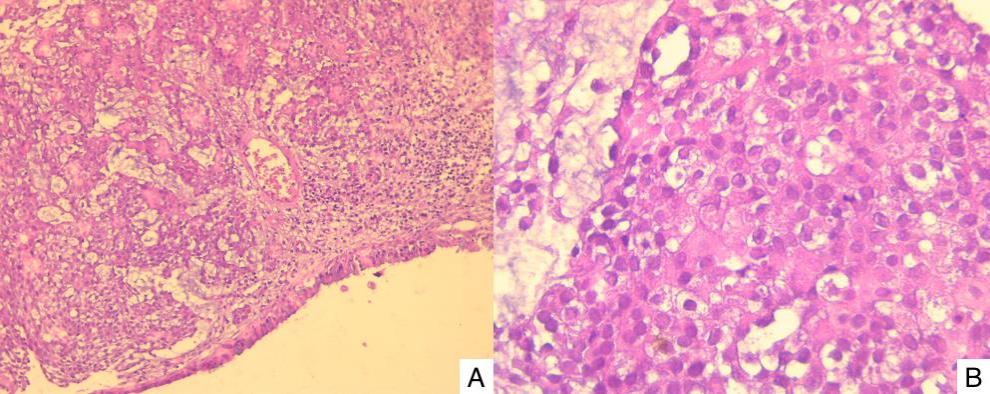
A) low power showing a proliferation of variably sized nests separated by thick mucoid stroma (H&E x100); B) high power showing tumor cells with plasmacytoid morphology and frequent mitoses (H& E x400)

**Figure 3:**
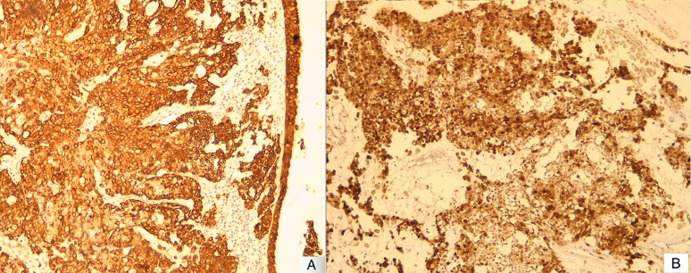
A) immunostaining for cytokeratin AE1/AE3; B) strong and diffuse expression for p16

Histology showed a tumor composed of variably sized nests, separated by thick mucoid stroma. The constituent cells were plasmacytoid with eosinophilic cytoplasm and mildly atypical hyperchromatic nuclei, and frequent mitoses. Lymphovascular and perineural invasion was not identified (Figure 2). Immunohistochemistry revealed that neoplastic cells were positive for cytokeratin AE1/AE3 (Figure 3) and negative for CK7, CK20, CD117, p40, p63, S100, synaptophysin and chromogranin. P16 showed strong and diffuse immunoreactivity (>70% nuclear and cytoplasmic staining) (Figure 3). HPV testing was unfortunately not performed due to the lack of means.

**Follow-up and outcomes:** unfortunately the patient was lost to follow up for one year, then he has reconsulted. A magnetic resonance imaging (MRI) was performed and showed an increase in the size of the ethmoid tumor (10 cm) with significant locoregional extension (stage T4b) and multiple submandibular lymphadenopathy. The patient was referred to the radiotherapy department.

## Discussion

HPV-related multiphenotypic sinonasal carcinoma (HMSC) is a rare distinct tumor of the sinonasal tract. To date there are less than 60 cases of HMSC in the literature including the largest series (49 cases) reported by Bishop *et al*. in 2017 [1,5]. Patient age ranged from 28 to 90 years with a mean of 54 years. Gender distribution was nearly equal, affecting 21 males and 28 females. Epistaxis and nasal obstruction were the most common presenting symptoms, ocular symptoms such as epiphora or exophthalmos are less frequent. The tumor sizes ranged from 0.7 to 8.5 cm. In all cases the tumor developed in the sinonasal tract, 57% are confined to the nasal cavity, 10% confined to the paranasal sinuses and 33% localized in nasal cavity and sinonasal sinuses. Most patients (23 of 39) presented with early stage disease (T1, T2), whereas the remainder (17 of 39) presented with advanced stage (T3, T4) disease. The majority of patients (36 of 38) underwent surgery. However, one patient received chemoradiation alone and one patient received radiation therapy alone. During follow up period, six developed local recurrence, with two cases eventually developing distant metastasis, no lymph node metastases or tumor related deaths were reported [1].

Microscopically, the tumor firstly defined by Bishop *et al*. was described as a carcinoma with highly cellular proliferation of basaloid cells in solid nests with focal cribriform structures and cylindromatous microcystic spaces [3]. The most common architectural pattern was solid which was demonstrated in the present case report. Cribriform and tubular growth pattern were less frequent. The tumor cells showed multidirectional phenotypes including myoepithelial (basaloid), ductal (luminal) and squamous lines of differentiation [1,3]. This histologic diversity approved the terminology of “multiphenotypic”. Cell spindling, cytoplasmic clearing and plasmacytoid morphology have also been reported including our own case were tumor cells showed plasmacytoid morphology.

The squamous component generally took the form of atypical surface epithelium [10]. Many cases demonstrate bizarre squamous epithelial atypia, with randomly distributed, hyperchromatic, giant nuclei and variably prominent nucleoli, but in a few cases the atypical epithelium resembled squamous dysplasia that could be encountered in other head and neck sites. Today this surface involvement is not clear whether a truly premalignant process or secondary intraepithelial tumor extension is. However, the atypical epithelium was observed in 69% of cases, thereby some cases lack of this surface involvement as well as our case [1].

Other newly recognized features included cellular anaplasia, an inverted growth pattern with fibrovascular cores, squamous differentiation within the invasive tumor, hemangiopericytoma-like vasculature and sarcomatoid features including chondro-osseous differentiation were recently described by Bishop *et al*. in 2017 [1]. By immunohistochemistry, both ductal and basaloid cells are positive for cytokeratin (AE1/AE3) and the expression is stronger in the ductal cells than in the luminal basaloid cells. The ductal cells typically express c-kit and the basaloid cells typically express myoepithelial markers, such as S100, p63, p40, calponin and smooth muscle actin [11].

As suggested by the literature, HMSC is characterized by a salivary gland tumor-like appearance and overlying surface dysplasia. The highlight of our case that the salivary gland component of our tumor did not show morphologic and immunophenotypic features that support the biphasic differentiation of ductal and myoepithelial cells, there were no ducts or cribriform structures surrounded by basaloid cells and the myoepithelial immunohistochemical markers were negative. Our tumor demonstrated variably sized nests and regular surface epithelium with negative immunostaining of p40 and p63, which could exclude the diagnostic of squamous cell carcinoma (SCC). Strong and diffuse immunoreactivity of p16 was also the diagnostic clue of HMSC. By definition HMSC is associated with high-risk HPV infection, P16 is a useful immunohistochemical marker, that all cases reported in the literature are positive for P16 with strong and diffuse immunoreactivity as well our case. HPV testing by ribonucleic acid (RNA) in situ hybridization or polymerase chain reaction (PCR) are the best methods to confirm HPV infection and to establish the diagnostic of HMSC. HPV type 33 is the most common HPV genotype associated with HMSC, followed by rare cases of HMSC associated with HPV type 35, 16, 56 and 52 [1,6-8]. SOX10 is a transcription factor that was described as a diagnostic nuclear marker of melanocytic and nerve sheath tumors. Recently, SOX10 positivity has also been reported in ACC and other salivary gland tumors [12].

More recently, in 2018, a study by Hsieh *et al*. has shown that HMSC cells are diffusely positive for SOX10, whereas the overlying atypical squamous epithelium and the squamous cell carcinoma (SCC) are negative for SOX10. This study concluded that SOX10 is a useful immunohistochemical marker for HMSC and especially to differentiate HMSC from HPV-related squamous cell carcinoma (SCC) [13]. However, Rooper *et al*. demonstrated that SOX10 is positive in 83% of basaloid variant of HPV-related SCC [12]. As such, the usefulness of SOX10 to distinguish HMSC and SCC, particularly basaloid variant, is very limited. This marker also cannot be used to differentiate HMSC from ACC. It has been established that ACC demonstrate fusions involving proto-oncogenes MYB or MYBL1, by consequence the majority of ACC showed overexpression of the MYB protein [14,15]. However, a recent study by Shah *et al*. concluded that MYB protein has limited utility in separating adenoid cystic carcinoma from HMSC. Indeed, more than half of HMSC cases (6 of 10) having moderate to strong intensity staining with MYB protein, despite consistently lacking MYB rearrangements [16].

## Conclusion

HMSC does not always closely resemble adenoid cystic carcinoma and can resemble squamous cell carcinoma or other salivary tumor types. The diagnosis should be considered for any unusual appearing carcinoma, especially if it resembles a salivary gland tumor. Finally, awareness of this newly recognized entity and judicious use of immunohistochemistry is imperative in avoiding an erroneous diagnosis, and hence guiding accurate patient management.
